# Prophylactic infusion of allogeneic double-negative T cells as immune modulators to prevent relapse in high-risk AML patients post-Allo-HSCT: a phase I trial

**DOI:** 10.1186/s40164-025-00680-1

**Published:** 2025-07-02

**Authors:** Guangyu Sun, Xingchi Chen, Tianzhong Pan, Kaidi Song, Haicun Xie, Meijuan Tu, Xiang Wan, Wen Yao, Yaxin Cheng, Ziwei Zhou, Dongyao Wang, Yongsheng Han, Baolin Tang, Liming Yang, Xiaoyu Zhu

**Affiliations:** 1 Department of Hematology, The First Affiliated Hospital of USTC, Division of Life Sciences and Medicine, University of Science and Technology of China, Hefei, Anhui China; 2 Anhui Provincial Key Laboratory of Blood Research and Applications, Hefei, China; 3 Blood and Cell Therapy Institute, Division of Life Sciences and Medicine, University of Science and Technology of China, Hefei, China; 4 Ruichuang Biotechnology Co., Ltd, Shaoxing, China

**Keywords:** Double negative T cell, Acute myeloid leukemia, Allogeneic hematopoietic stem cell transplantation, Relapse, Adoptive cellular therapy, Immune modulator

## Abstract

Relapse remains a major challenge for high-risk acute myeloid leukemia (AML) patients following allogeneic hematopoietic stem cell transplantation (allo-HSCT). In our first-in-human Phase I trial (ChiCTR-1900022795), we have demonstrated that third-party donor-derived double-negative T cells (DNTs) are safe and effective for treating relapsed AML. This Phase I study aims to further evaluate the safety and efficacy of allo-DNTs in preventing relapse in AML patients post-allo-HSCT. Six high-risk AML patients received three infusions of off-the-shelf allo-DNTs at one-month intervals, administered 60 to 100 days post-allo-HSCT without lymphodepleting chemotherapy. No dose-limiting toxicity, DNT-related graft-versus-host disease (GvHD), or severe cytokine release syndrome (CRS) occurred. With a median follow-up of 20.9 months (range: 11.4–24.6), four patients (66.7%) remained in minimal residual disease (MRD)-negative complete remission (CR), with recurrence-free survival exceeding 24 months. Patients in remission showed increased CD8⁺ and CD4⁺ T cells, total DNTs, and higher frequencies of granzyme-secreting T cells, which were absent in relapsed patients. In vitro, co-culturing AML patient CD8⁺ T cells with allo-DNTs upregulated granzyme B and interferon-γ expression, indicating CD8⁺ T cell activation. These findings suggest that allogeneic DNT immunotherapy is a safe, promising strategy to prevent relapse in high-risk AML patients post-allo-HSCT by combining intrinsic antitumor activity with immune modulation.

**Supplementary Information:**

The online version contains supplementary material available at 10.1186/s40164-025-00680-1.

**To the editor**

Allogeneic hematopoietic stem cell transplantation (allo-HSCT) is a standard treatment for high-risk acute myeloid leukemia (AML), yet about 40% of patients experience relapse [1–5]. Post-transplant interventions offer limited efficacy and carry high risks, including graft-versus-host disease (GvHD) and high treatment-related mortality [6]. Allogeneic double-negative T cells (allo-DNTs, CD3⁺CD4⁻CD8⁻), though rare (1–5% of peripheral leukocytes), show potent graft-versus-leukemia (GVL) activity without GvHD [7]. Our Phase I clinical trial (ChiCTR-1900022795) demonstrated that DNT therapy induced 50% complete remission (CR) rate in relapsed AML patients post-HSCT [8]. Here, we report a prophylactic trial of allo-DNT infusion in six AML patients post-allo-HSCT, demonstrating a favorable safety profile and early evidence supporting the potential of allogeneic DNT cell therapy as an immune-modulating approach to prevent relapse in high-risk AML patients following allo-HSCT.

## Characterization of patients and DNTs

Six patients (median age 44 years; range 27–62) were enrolled (Additional file 1). The median prior treatment lines were 3 (range 1–8) before allo-HSCT (Table 1). Three patients were refractory/relapsed AML, one of which also possessed pre-transplant progressive disease and high-risk genetic mutations (IDH2 and TP53), and the other one had disease progression pre-transplantation. Two patients suffered history of myelodysplastic syndrome (MDS), one of which also had IDH2 mutation-positive pre-transplantation. The remaining one was multiparameter flow cytometry (MFC) MRD-positive pre-transplantation. One patient received haplo-HSCT, others underwent umbilical cord blood transplantation. Third-party allo-DNTs were administered without lymphodepletion, starting at a median of 92.5 days (range 64–100) post-HSCT. Each patient received three monthly infusions at escalating doses of 1 × 10⁸ and 1.5 × 10⁸ cells/kg.

**Table 1:** Clinical characteristics of the patients

Pt No.	Dose Level (/kg)	Age	Sex	Risk Profile		Prior treatment	Transplant type	Time between transplant and DNT infusion (days)	Pre-infusion level (count/ml)		
				2022 ELN Risk Category	High risk factors for recurrence				CD4^+^T	CD8^+^ T	DNT
01002	1 × 10^8^	62	F	Adverse	Refractory AML; Disease progression before allo-HSCT; Genetic high-risk factors (IDH2^+^ & TP53^+^)	Aza + Venetoclax→CR(MRD^+^)→Aza+ Venetoclax ×2→relapse/NR→haplo-HSCT	haplo-HSCT	92	111	0	4550
01003	1 × 10^8^	52	M	Adverse	History of MDS	MDS(IB-1)→secondary AML→Cladribine+ Ara-C + Venclexta→CR→UCBT	UCBT	100	245,386	690,142	26,433
01005	1 × 10^8^	27	F	Favorable	Refractory AML	IA→CR1→IA→Ara-C×2→relapse→M + CLAG→CR→UCBT	UCBT	64	221,964	477,453	44,604
01007	1.5 × 10^8^	35	M	Adverse	MRD positive before allo-HSCT	IA→CR→IA→HHT + Ara-C→Ara-C×2→UCBT (CBFβ-MYH11^+^)	UCBT	93	3195	3901	111
01008	1.5 × 10^8^	35	M	Adverse	Refractory AML; Disease progression before allo-HSCT;	IA→CR1→IA→Ara-C×2→HHT + Ara-C→relapse→IDA + CLAG→NR→IDA + CLAG→NR→UCBT	UCBT	96	159,730	389,614	2285
01009	1.5 × 10^8^	53	F	Adverse	History of MDS; MRD positive before allo-HSCT	Venetoclax (IDH2 0.64%)→CR (IDH2^+^)→IA→Ara-C→MRD^+^ (IDH2^+^)→UCBT	UCBT	85	192,616	510,483	6472

Abbreviations: IDH2, Isocitrate Dehydrogenase 2; haplo-HSCT, haploidentical hematopoietic stem cell transplantation; UCBT, umbilical cord blood transplantation; r/r AML, relapsed/refractory Acute Myeloid Leukemia; Aza, Azacitidine; Ara-C, Cytarabine; IA, Idarubicin (IDA) & Cytarabine; M + CLAG, Mitoxantrone & Cladribine & Cytarabine & G-CSF (granulocyte colony stimulating factor); CR, Complete remission; MDS-IB, Myelodysplastic syndromes with increased blasts; M, Male; F, Famale; NR, Non-remission; MRD, minimal residual disease; Pt, patient; ELN, European leukemia net; DNT, double negative T cell

### Safety profiles

**Fig. 1:**
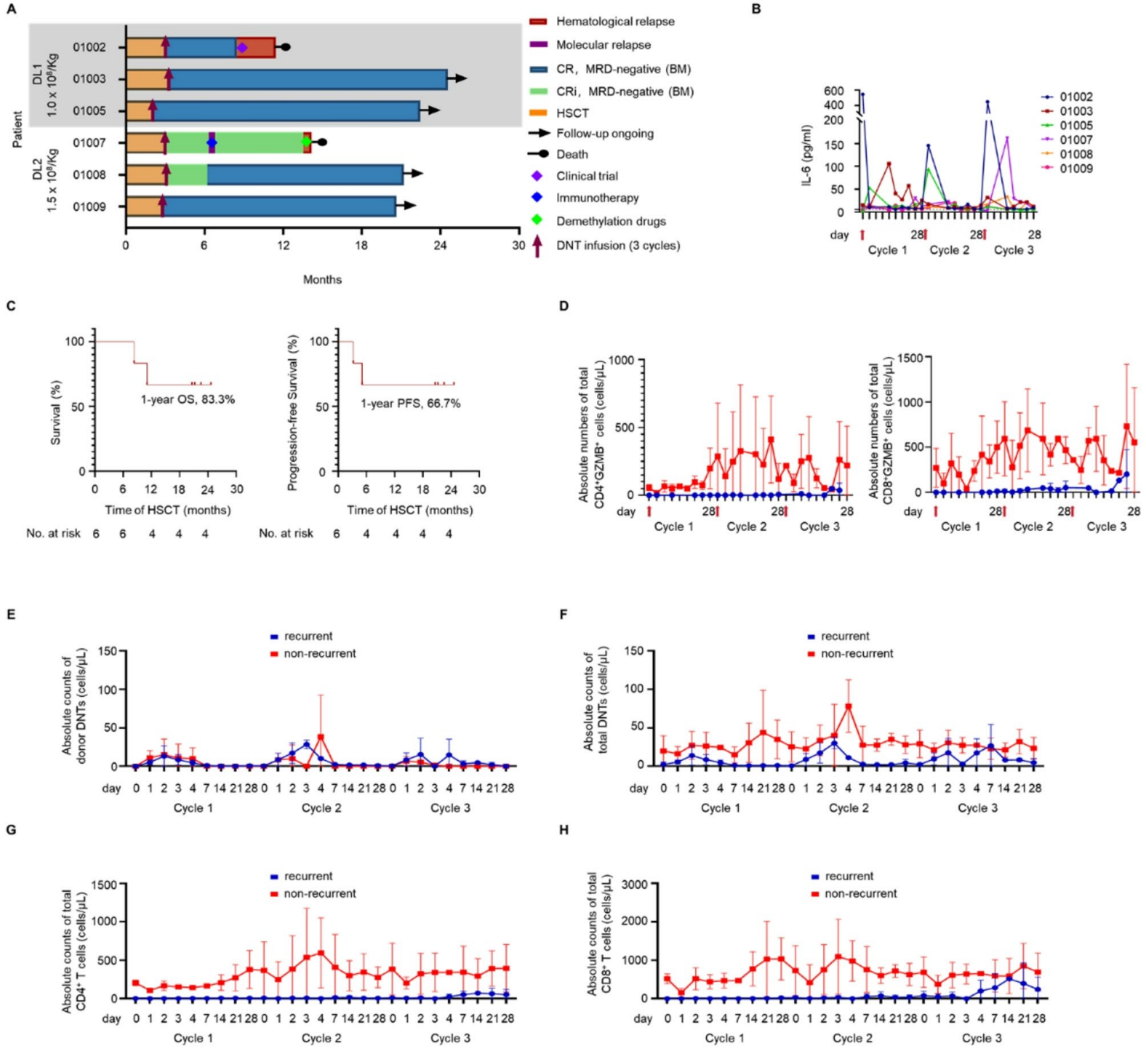
Clinical outcome and kinetics of peripheral blood biomarkers following prophylactic infusion of allo-DNTs. (**A**) Swimmer plots showing the clinical outcomes of six patients after three doses of allo-DNTs treatment. CRi: incomplete CR. (**B**) Measurement of plasma IL-6. (**C**) Kaplan–Meier curves showing predicted 1-year OS and 1-year PFS post HSCT. (**D**) Total absolute number of GZMB^+^ CD4^+^ T and CD8^+^ T cells. (**E-H**) Absolute counts of donor-DNTs (**E**), total DNTs (**F**), recipient-CD4^+^ T cells (**G**), and recipient -CD8^+^ T cells (**H**)

Mild infusion reactions (fever, headache, hypertension) occurred in 72.2% (13/18) of infusions, resolving within 24 h. Most patients experienced transient IL-6 and IL-10 elevation (Fig. 1B, Additional file 2: Figure S1). One patient developed mild cytokine release syndrome six days post-first infusion and resolved spontaneously within a week; another reported mild joint pain and resolved without intervention. A third developed toxic erythema on the lower limbs 34 days post-infusion, improving with topical glucocorticoids. Patient #01003 experienced mild chronic GvHD during the second infusion, which resolved without intervention and was thought to be unrelated to DNT infusion. No cases of DNT-related acute GvHD or neurotoxicity were observed (Additional file 2: Table S1).

### Clinical outcomes

As of May 1, 2025, with a median follow-up of 20.9 months (range: 11.4–24.6), four patients (66.7%) maintained MRD-negative CR without further treatment (Additional file 2: Table S2), with the longest recurrence-free survival exceeding 24 months (Fig. 1A). The 1-year OS and progression-free survival (PFS) were 83.3% and 66.7%, respectively, and the 1-year relapse incidence was 33.3% (95% CI: 9.6–80.5%) (Fig. 1C), with no non-relapse mortality. Patient #01007 experienced molecular relapse 28 days post-final infusion, achieved MRD-negativity with interferon α2b, but relapsed and died 14.2 months post-HSCT. Patient #01002, with TP53 and IDH2 mutations, complex karyotype (43–46, XX, -3, del (5) (q11q31)), del(7q10), -17), experienced pre-transplant disease progression, relapsed 8.1 months post-HSCT and died.

### PK and immunomodulatory functions of allo-DNTs in patients

Donor-derived DNTs were detected in peripheral blood 1–4 days post-infusion, persisting up to 28 days and, in two non-recurrent patients, up to 360 days (Fig. 1E, Additional file 2: Table S3). Non-recurrent patients exhibited higher cytokines levels and greater expansion of total and recipient-derived DNTs, CD4⁺, and CD8⁺ T cells post-infusion (Fig. 1F-H, Additional file 2: Figure S1). They had higher effector memory T cells (T_em_) proportions and granzyme expression (Fig. 1D, Additional file 2: Figure S2). However, cytokine and immune cells levels were relatively low in recurrent patients. In vitro, co-culturing patient-derived CD8⁺ T cells with allo-DNTs upregulated IFN-γ and GZMB expression, immune activation-associated cytokines and pathways (Additional file 2: Figure S3, Table S4).

In conclusion, despite our small sample size and limited follow-up, this study highlights unique advantages of allo-DNT therapy over existing post-HSCT interventions. Allo-DNT infusions, administered without lymphodepleting preconditioning, minimize treatment-related toxicity and, as an off-the-shelf product, offer a ready-to-use option for relapse prevention. Additionally, DNTs possess a dual immune-regulatory function by enhancing the indirect GVL effect through CD8⁺ T cell activation while mitigating GvHD risk. Our findings underscore the promising potential of DNT cell infusions to prevent relapse in high-risk AML patients post-transplant.

## Electronic supplementary material

Below is the link to the electronic supplementary material.

Supplementary Material 1

Supplementary Material 2

## Data Availability

No datasets were generated or analysed during the current study.

## Abbreviations

AML: Acute myeloid leukemia
allo-HSCT: Allogeneic hematopoietic stem cell transplantation
UCBT: Umbilical cord blood transplantation
DNTs: Double negative T cells
DLT: Dose-limiting toxicity
GvHD: Graft-versus-host disease
CRS: Cytokine release syndrome
CR: Complete remission
MRD: Measurable residual disease
OS: Overall survival
PFS: Progression-free survival
DLI: Donor lymphocyte infusion
GVL: Graft-versus-leukemia
PK: Pharmacokinetics
T_em_: Effector memory T cells
IFN-γ: Interferon-gamma
GZM: Granzyme
MDS: Myelodysplastic syndrome
IDH2: Isocitrate Dehydrogenase 2
ELN: European leukemia net
