# Supplementary material for: Prophylactic infusion of allogeneic double-negative T cells as immune modulators to prevent relapse in high-risk AML patients post-Allo-HSCT: a phase I trial
Source: Exp Hematol Oncol. 2025 Jul 2;14:90. doi: 10.1186/s40164-025-00680-1 (PMC12224462; doi:10.1186/s40164-025-00680-1)
Supplement: Supplementary file 1 — Supplementary Material 1 [file 40164_2025_680_MOESM1_ESM.docx]

**SYNOPSIS**

**Title：Allo-DNT Cells for the Prevention of Relapse in AML Patients After Allo-HSCT**

**Objective**

To evaluate the safety and tolerability of healthy donor-derived double negative T cells (DNTs) infusion in acute myeloid leukemia (AML) patients after allogeneic hematopoietic stem cell transplantation (allo-HSCT).

**Trial Design**

This is a phase 1/11 study to assess the safety and efficacy of healthy donor-derived DNTs in AML patients after allo-HSCT. The trial is divided into two parts: Part A is a dose escalation trial with two dose groups (1.0×10^8 cells/kg, 1.5×10^8 cells/kg at day 0, day 28 and day 56), with 6-12 patients planned to be enrolled. Part B is a dose-expansion randomized controlled trial in which 40-60 patients will receive RC1012 infusions at recommended phase II dose (RP2D) levels.

**Patient Population**

AML patients after allo-HSCT are eligible for enrollment.

**Sample Size**

We plan to recruit 6-12 patients.

**Main Inclusion and Exclusion Criteria**

**Patient inclusion criteria**

- Voluntarily sign an ICF and expect to complete the study procedures for follow-up examinations and treatment.
- Aged 18 to 70 years (including cut-offs), regardless of gender.
- Subject must be diagnosed with AML according to World Health Organization (WHO) criteria (2016).
- The subject has received an allo-HSCT within 60-100 days, the percentage of malignant primitive cells in the bone marrow is < 5% after HSCT and STR-PCR shows complete donor chimerism.
- The subject has one of the following high-risk factors for relapse after allo-HSCT: (1) Failure to achieve remission after two courses of induction chemotherapy. (2) Prior history of Myelodysplastic Syndromes (MDS) or Myeloproliferative Neoplasm (MPN). (3) High leukocytes (≥100×10^9/L) combined with Central Nervous System Leukemia (CNSL); (4) Positive Minimal Residual Disease (MRD) before HSCT; (5) Non-remission or disease progression prior to HSCT; (6) Subject with cytogenetic high-risk factors (except that who can be treated with targeted drugs).
- The subject has recovered from the toxicity of the prior treatment, i.e., CTCAE toxicity grade <2 (unless the abnormality is tumor-related).
- ECOG score 0 to 1.
- With appropriate organ function.

**Patient exclusion Criteria**

- Subject is confirmed to have morphological relapse of leukemia or positive MRD after allo-HSCT.
- Subject with extramedullary infiltration of leukemia.
- Suffer from other malignancies within 5 years prior to screening, except adequately treated carcinoma in situ of the cervix, basal cell or squamous epithelial cell skin cancer, post-radical thyroid cancer, and post-radical ductal carcinoma in situ.
- Has severe respiratory disease.
- A previous history of a definite neurological or psychiatric disorder, including epilepsy or dementia.
- Evidence of active central nervous system invasion or cranial neuropathy.
- Patients with positive hepatitis B surface antigen (HBsAg) or hepatitis B core antibody (HBcAb) and peripheral blood hepatitis B virus (HBV) DNA titration assay not within the normal reference range, positive hepatitis C virus (HCV) antibody and peripheral blood HCV RNA ,positive for human immunodeficiency virus (HIV), or positive for cytomegalovirus (CMV) DNA, or positive syphilis test.
- Subject who is allergic to the excipients of RC1012 injection or other drugs recommended in the study protocol (e.g., tolimumab, etc.).
- Serious cardiac disease, including but not limited to severe arrhythmia, unstable angina, massive heart attack, New York Heart Association class III or IV cardiac insufficiency, refractory hypertension.
- Persons who have previously received an organ transplant or are preparing to receive an organ transplant (except for HSCT).
- Subject who has received other maintenance therapy drugs after HSCT or who wish to receive other maintenance therapy.
- Subject with the presence of acute GvHD of degree III to IV or extensive chronic GvHD.
- Active neurological autoimmune or inflammatory diseases (e.g. Guillain-Barre Syndrome (GBS), Amyotrophic lateral sclerosis (ALS)).
- Clinically significant active cerebrovascular disease (e.g. cerebral oedema, Posterior Reversible Encephalopathy Syndrome (PRES)).
- Subject with a life expectancy of less than 3 months.
- Subject has been involved in other clinical studies within 3 months prior to screening.
- Subject in the judgement of the investigator and/or clinical criteria, is contraindicated to any study procedure or have other medical conditions that may place them at unacceptable risk.

**Donor inclusion criteria**

- Age ≥ 18 years old.
- Healthy person who meet the requirements of blood donation in central blood station.
- There is no disease that can be transmitted through blood within 30 days before blood collection (HIV, HBsAg, HBcAb, HCV antibody, syphilis serological test, CMV IgM, and EBV IgM all negative).
- The cytotoxicity of DNTs to at least three myeloid leukemia cell lines (OCI-AML3, MV 4-11, K562, U937, etc.) (target to efficiency ratio 4:1) ≥ 20%, or to patient primary AML cells ≥ 10%.

**Donor exclusion criteria**

- There are chronic diseases that need to be treated, such as hypertension, diabetes, heart disease, thyroid disease and liver and kidney insufficiency.
- Bone marrow diseases.
- Pregnancy.
- Systemic glucocorticoids must be used before peripheral blood taking.
- Paroxysmal psychosis.
- Poison and drug addiction.

**Trial Schema**


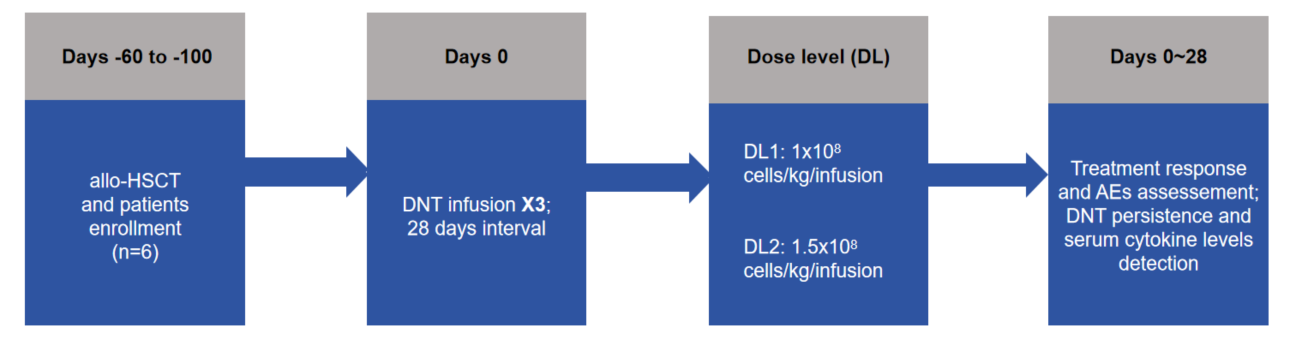


**TABLE OF CONTENTS**

**1 BACKGROUND**1

**2 OBJECTIVES**3

2.1 Primary Objectives3

2.2 Secondary Objective4

**3 TRIAL DESIGN**5

**4 ELIGIBILITY**5

4.1 Subject Eligibility 5

4.1.1 Inclusion Criteria 6

4.1.2 Exclusion Criteria7

4.1.3 Criteria For Removal From Study8

4.2 Donor Eligibility8

4.2.1 Inclusion Criteria8

4.2.2 Exclusion Criteria8

**5 TREATMENT PLAN**9

5.1 Ex vivo expansion of DNTs and Infusion9

5.2 Supportive Treatment9

5.3 Assessment10

5.4 Follow-up11

**6 TREATMENT RESPONSE/OUTCOME ASSESSMENT** 11

6.1 Main Outcome Measurement11

6.2 Secondary Outcome Measurement11

6.3 Safety Measurement12

6.4 Response Assessment12

6.5 Relapse13

6.6 Survival13

**7 ADVERSE EVENT REPORTING**13

7.1 Definitions13

7.2 Attribution 14

7.3 Procedures for AE and SAE Recording and Reporting14

7.4 Follow-up of AE14

7.5 Gestation15

**8 QUALITY MANAGEMENT**15

8.1 Investigators15

8.2 Clinical Monitors15

8.3 Standardized Operation15

8.4 Laboratory Examination16

**9 DATA HANDLING AND RECORD KEEPING**16

9.1 Data Collection16

9.2 Data Retention16

**10 SAMPLE SIZE** 16

**11 REFERENCE**17

**12 APPENDIX**18

1. **BACKGROUND**

Acute myeloid leukemia (AML) is a heterogeneous hematologic malignancy characterized by abnormal proliferation, differentiation, and clonal expansion of hematopoietic stem/progenitor cells that invade the bone marrow, blood, and extramedullary tissues. Allogeneic hematopoietic stem cell transplantation (allo-HSCT) is currently the primary curative approach for relapsed/refractory AML, but more than 30% of AML patients experience post-transplant relapse. Relapsed patients have a poor prognosis, with a two-year overall survival rate of less than 15%, and relapse is a major cause of post-transplant mortality(1). High-risk factors for relapse include complex karyotype, TP53 gene mutations, failure to achieve complete remission after two cycles of induction chemotherapy, and minimal residual disease (MRD) positivity before transplantation. Despite allo-HSCT being the primary curative approach for relapsed/refractory AML, more than 30% of patients relapse after transplant. The prognosis for relapsed patients remains poor, with a two-year overall survival rate of less than 15%, making relapse the leading cause of post-transplant mortality(2). So far, the main strategies for preventing post-transplant relapse include optimizing conditioning regimens, improving GvHD prevention protocols, post-transplant maintenance therapy, and prophylactic donor lymphocyte infusion (DLI)(3).

DNT cells are a subset of mature T lymphocytes, constituting about 1-5% of peripheral blood mononuclear cells (PBMC) in mice, rats, and humans. DNT cells express CD3 and γδ-TCR but do not express CD4, CD8, or NK cell markers, nor do they bind to the invariant natural killer T cell (iNKT)-specific αGalCer-loaded CD1d tetramer(4). Therefore, they are distinct from conventional T cells, NK cells, and NKT cells. Zhang’s laboratory at the University of Toronto was the first in the world to report the characteristics of antigen-specific DNT cells with transplant immune tolerance, and they conducted extensive studies on how these cells differ from traditional CD4^+^ or CD8^+^ T cells. Notably, transplantation of allogeneic DNT cells into mice not only did not induce graft-versus-host disease (GvHD) but also suppressed GvHD caused by allogeneic CD4^+^ and CD8^+^ T cells(5).

To determine the anti-leukemic function of DNT cells, RuiChuang Biotech evaluated the cytotoxicity of DNT cells expanded in vitro from healthy donors (HD) against blast cells derived from the peripheral blood of AML patients. The study found that after co-culturing these cancer cells with DNT cells expanded from HDs, DNT cells were able to kill AML cells in over 70% of the patients, exhibiting a dose-dependent killing effect. Additionally, the anti-leukemic activity mediated by DNT cells was higher than that of NK cells and CD8^+^ T cells. Using a patient-derived xenograft (PDX) model for AML, Zhang’s lab discovered that, compared to the PBS-treated control group, a single infusion of allogeneic DNT cells from healthy donors significantly reduced the levels of CD45^+^CD33^+^ leukemic cells, previously engrafted into the bone marrow of mice from AML patients(6). Similar results were observed in experiments involving three additional infusions of AML blasts and three different HD-derived DNT cells expanded in vitro. A single infusion of RC1012 solution reduced the AML blast burden in the experimental mice, and two additional infusions of DNT cells administered every three days further decreased the leukemic burden, demonstrating that multiple infusions of DNT cells enhanced the therapeutic efficacy.

Allo-HSCT can induce a curative graft-versus-leukemia (GVL) effect, but it may also lead to other diseases or mortality due to donor T cells attacking the recipient’s normal cells and tissues. Previous studies have confirmed that the infusion of allogeneic mouse DNT cells into NSG mice does not cause GvHD and can suppress GvHD caused by the transplantation of CD4^+^ and CD8^+^ T cells from the same allogeneic source(7). Similar to mouse DNT cells, human DNT cells can also inhibit the in vitro proliferation of corresponding CD4^+^ and CD8^+^ T cells. In patients who received allo-HSCT, a higher frequency of DNT cells has been associated with reduced severity of GvHD, suggesting the potential therapeutic benefits of DNT cell treatment(8). To verify the effects of in vitro-expanded human DNT cells in a GvHD setting, DNT cells and/or PBMCs from different donors were intravenously infused into NSG mice, and the incidence of GvHD was observed. Consistent with previous reports, mice infused with human PBMCs developed severe acute GvHD, manifested by weight loss. Histological analysis of the PBMC-infused mice revealed acute GvHD lesions in multiple organs. However, in mice infused with an equivalent or fourfold higher number of DNT cells compared to PBMCs, although lymphocytic infiltration was observed, there was no weight loss, and no organ damage was found in the liver, lungs, or intestines. Even 50 days after DNT cell infusion, the mice still showed no signs of GvHD. In summary, these studies suggest the safety of RC1012 injections (with DNT cells as the main component).

Collectively, our data indicate that DNT cells is a novel off-the-shelf adoptive cellular therapy (ACT) against AML. Based on these preclinical data, this trial intends to enroll 6~12 patients to assess the safety and efficacy of increasing the dose of DNT cells in the prevention of AML patients after allo-HSCT.

1. **OBJECTIVES**

**2.1 Primary Objectives**

- Dose-Limiting Toxicity (DLT)

To evaluate the safety, tolerability, and determine the recommended dosage of allo-DNT cell therapy for AML subjects after allo-HSCT. Time Frame: Up to 28 days.

- Maximum Tolerated Dose (MTD)

MTD was the highest dose for DLT in ≤1/6 subjects. Time Frame: Up to 28 days.

- Incidence of abnormalities: Incidence of abnormalities in AE/SAE/laboratory tests/electrocardiograms/vital signs. Time Frame: Up to 28 days.

**2.2 Secondary Objectives**

- Pharmacokinetics (PK) indicator (Cmax)

The peak concentration of allo-DNT cells amplified in the peripheral blood (Cmax, detected by Flow Cytometry). Time Frame: Up to 2 years.

- Pharmacokinetics (PK) indicator (AUC)

Allo-DNT cells blood concentrations will be measured at different time points to evaluate the area under the curve (AUC). (AUC, detected by Flow Cytometry). Time Frame: Up to 2 years.

- Pharmacokinetics (PK) indicator (Tmax)

Allo-DNT cells blood concentrations will be measured at different time points to evaluate the peak time (Tmax) in peripheral blood. Tmax is defined as the time to reach the highest concentration (Tmax, detected by Flow Cytometry). Time Frame: Up to 2 years.

- Pharmacokinetics (PK) indicator (T1/2)

Allo-DNT cells blood concentrations will be measured at different time points to evaluate the elimination half-life in hours (T1/2). T1/2 is defined as the time point when the concentration of allo-DNT reaches half of maximum in a patient’s peripheral blood (T1/2, detected by Flow Cytometry). Time Frame: Up to 2 years.

- Recurrence rate at 6 months

Record the proportion of patients with recurrence in the study. Time Frame: Up to 6 months.

- Relapse-free survival (RFS)

Relapse-free survival is the time from study enrollment until documented disease relapse, or death from any cause. Time Frame: Up to 2 years.

- GvHD-free and relapse-free survival (GRFS)

GRFS is the time from study enrollment until documented disease relapse, or death, or GvHD occur from any cause. Time Frame: Up to 2 years.

- 2-year overall survival

From the date of entry into the clinical study until death from any cause. Time Frame: Up to 2 years.

1. **TRIAL DESIGN**

The trial is divided into two parts: Part A is a dose escalation trial with two dose groups (1.0×10^8 cells/kg, 1.5×10^8 cells/kg at day 0, day 28 and day 56), with 6-12 patients planned to be enrolled. Part B is a dose-expansion randomized controlled trial in which 40-60 patients will receive RC1012 infusions at recommended phase II dose (RP2D) levels. Patients at the end of the infusion will continue to be followed up for a total of 2 year.

1. **ELIGIBILITY**
   1. **Subject Eligibility**
      1. **Inclusion Criteria**

- Voluntarily sign an ICF and expect to complete the study procedures for follow-up examinations and treatment.
- Aged 18 to 70 years (including cut-offs), regardless of gender.
- Subject must be diagnosed with AML according to World Health Organization (WHO) criteria (2016).
- The subject has received an allo-HSCT within 60-100 days, the percentage of malignant primitive cells in the bone marrow is < 5% after HSCT and STR-PCR shows complete donor chimerism.
- The subject has one of the following high-risk factors for relapse after allo-HSCT: (1) Failure to achieve remission after two courses of induction chemotherapy. (2) Prior history of Myelodysplastic Syndromes (MDS) or Myeloproliferative Neoplasm (MPN). (3) High leukocytes (≥100×10^9/L) combined with Central Nervous System Leukemia (CNSL); (4) Positive Minimal Residual Disease (MRD) before HSCT; (5) Non-remission or disease progression prior to HSCT; (6) Subject with cytogenetic high-risk factors (except that who can be treated with targeted drugs).
- The subject has recovered from the toxicity of the prior treatment, i.e., CTCAE toxicity grade <2 (unless the abnormality is tumor-related).
- ECOG score 0 to 1.
- With appropriate organ function:
- • Glutathione aminotransferase (AST) ≤ 3 times the upper limit of normal (ULN);
- • Glutamic aminotransferase (ALT) ≤ 3 times ULN;
- • Total bilirubin ≤ 1.5 times ULN, unless the patient has documented Gilbert syndrome. Patients with Gilbert-Meulengracht syndrome with total bilirubin ≤ 3.0 times ULN and direct bilirubin ≤ 1.5 times ULN may be included;
- • Serum creatinine ≤ 1.5 times ULN or a creatinine clearance ≥ 60 ml/min;
- • Hemoglobin ≥ 80 g/L or hemoglobin maintained at that level following transfusion;
- • International Normalized Ratio (INR) ≤ 1.5 times ULN and Activated Partial Thromboplastin Time (APTT) ≤ 1.5 times ULN;
- • Absolute neutrophil count (ANC) ≥ 1.5 x 10^9/L.
- • A platelet count ≥ 50 x 10^9/L or a platelet count maintained at that level following a platelet transfusion;
- • Left ventricular ejection fraction (LVEF) ≥ 45%.
- Female patients with childbearing potential should have a negative pregnancy test during the screening period. Any male and female patients of childbearing potential must agree to use an effective contraception method for at least six months from the time that they sign the informed consent form until the end of the cell infusion. Female patients without childbearing potential (meeting at least 1 of the following criteria) is described below.
- • Have undergone a hysterectomy or bilateral oophorectomy;
- • Medically recognized as ovarian failure;
- • Medically recognized as post-menopausal (at least 12 consecutive months of menopause without pathological or physiological cause).
  - 1. **Exclusion Criteria**
- Patients with active grade II to IV acute GvHD or extensive chronic GvHD.
- Subject is confirmed to have morphological relapse of leukemia or positive MRD after allo-HSCT.
- Subject with extramedullary infiltration of leukemia.
- Suffer from other malignancies within 5 years prior to screening, except adequately treated carcinoma in situ of the cervix, basal cell or squamous epithelial cell skin cancer, post-radical thyroid cancer, and post-radical ductal carcinoma in situ.
- Has severe respiratory disease (previous or combined history of severe interstitial lung disease, severe chronic obstructive pulmonary disease, severe pulmonary insufficiency, symptomatic bronchospasm).
- A previous history of a definite neurological or psychiatric disorder, including epilepsy or dementia.
- Evidence of active central nervous system invasion or cranial neuropathy.
- Patients with positive hepatitis B surface antigen (HBsAg) or hepatitis B core antibody (HBcAb) and peripheral blood hepatitis B virus (HBV) DNA titration assay not within the normal reference range, positive hepatitis C virus (HCV) antibody and peripheral blood HCV RNA ,positive for human immunodeficiency virus (HIV), or positive for cytomegalovirus (CMV) DNA, or positive syphilis test.
- Subject who is allergic to the excipients of RC1012 injection or other drugs recommended in the study protocol (e.g., tolimumab, etc.).
- Serious cardiac disease, including but not limited to severe arrhythmia, unstable angina, massive heart attack, New York Heart Association class III or IV cardiac insufficiency, refractory hypertension.
- Persons who have previously received an organ transplant or are preparing to receive an organ transplant (except for HSCT).
- Subject who has received other maintenance therapy drugs after HSCT or who wish to receive other maintenance therapy.
- Subject with the presence of acute GvHD of degree III to IV or extensive chronic GvHD.
- Active neurological autoimmune or inflammatory diseases (e.g. Guillain-Barre Syndrome (GBS), Amyotrophic lateral sclerosis (ALS)).
- Clinically significant active cerebrovascular disease (e.g. cerebral oedema, Posterior Reversible Encephalopathy Syndrome (PRES)).
- Subject with a life expectancy of less than 3 months.
- Subject has been involved in other clinical studies within 3 months prior to screening.
- Subject in the judgement of the investigator and/or clinical criteria, is contraindicated to any study procedure or have other medical conditions that may place them at unacceptable risk.
  - 1. **Criteria for Removal from the Study**
- The occurrence of intolerable toxicity during infusion prevents the subject from continuing RC1012 injection treatment.
- The subject may choose to withdraw from the clinical trial at any time by informing the researcher.
- Hematologic relapse of the patient’s disease occurs, and the researcher evaluates that cell infusion is not appropriate.
- The subject experiences death, withdrawal of informed consent, or is lost to follow-up during the trial.
- Other situations where the researcher deems cell infusion inappropriate.
  1. **Donor** **Eligibility**
     1. **Inclusion Criteria**
- Age ≥ 18 years old.
- Healthy person who meets the requirements of blood donation in central blood station.
- There is no disease that can be transmitted through blood within 30 days before blood collection (HIV, HBsAg, HBcAb, HCV antibody, syphilis serological test, CMV IgM, and EBV IgM all negative).
- The cytotoxicity of DNTs to at least three myeloid leukemia cell lines (OCI-AML3, MV 4-11, K562, U937, etc.) (target to efficiency ratio 4:1) ≥ 20%, or to patient primary AML cells ≥ 10%.
  - 1. **Exclusion Criteria**
- There are chronic diseases that need to be treated, such as hypertension, diabetes, heart disease, thyroid disease and liver and kidney insufficiency.
- Bone marrow diseases.
- Pregnancy.
- Systemic glucocorticoids must be used before peripheral blood taking.
- Paroxysmal psychosis.
- Poison and drug addiction.

1. **TREATMENT PLAN**
   1. **Ex vivo expansion of DNTs and Infusion**

According to the established expansion method (Patent number: PCT/CA2006/001870), DNTs will be expanded *ex vivo* under GMP conditions. DNTs will be assessed for purity and microorganism and endotoxin contamination before infusion. After enrollment, patients will receive once a month with total three times infusion of DNTs product at escalating doses of 1×10^8^, or 1.5×10^8^/kg. Subjects' vital signs will be assessed, and pulse oximetry will be done during the infusion.

- 1. **Supportive Treatment**

All medication treatments administered to the subject from the onset of AML until the end of this study (including RFS and OS follow-up periods) should be documented. This includes prescription drugs, over-the-counter medications, traditional Chinese medicine, Chinese patent medicines, and herbal preparations. Additionally, the subject’s medical history for other diseases from the time of signing the informed consent until the end of the treatment period (28 days after the last cell infusion) must also be recorded. Except for the prohibited medications specified in the study protocol, all other medications may continue to be used. From the initiation of RC1012 injection infusion until hematologic relapse or a change in the treatment regimen, unless otherwise specified in the protocol, the subject is not allowed to use the following treatments:

- Anti-tumor therapies (including traditional Chinese medicine, Chinese patent medicines, and herbal preparations with anti-tumor properties);
- Corticosteroids, as they may interfere with the activity of the product, should be avoided as a preventive measure. However, they may be used if necessary, such as in cases of CRS (Cytokine Release Syndrome);
- Any other medications that could affect the investigator's ability to assess efficacy or subject safety.
  1. **Assessment**
- Clinical Assessment: Clinical assessment including vital signs (e.g., blood pressure, pulse and temperature) and complications should be assessed every day during the cycle of therapy.
- Hematological examination: should be assessed 2-3 times per week. Blood routine should be monitored daily when ANC ＜ 1.0×10^9^/L and (or) platelet count ＜ 20×10^9^/L.
- Urine routine will be tested before each cycle of therapy and be retested after treatment according to the condition.
- Stool Routine: Stool routine will be tested before each cycle of therapy. Stool routine should be retested in time when patient have diarrhea, and bacterial and fungal culture should be carried out to exclude infectious diarrhea.
- Biochemistry: Biochemistry will be assessed before infusion and at least weekly for each cycle of therapy.
- Serum Cytokine Levels: Cytokines will be measured before infusion of DNTs, then 6, 24, 48 hours, 5d, 7d after each DNT infusion, and 14d, 21d, 28d after the 3^rd^ infusion. Serum cytokines included interleukin-6 (IL-6), interferon (IFN)-γ, IL-8, IL-10, tumor necrosis factor (TNF)-α, macrophage inflammatory protein (MIP)-1α, MIP-1β, monocyte chemoattractant protein (MCP-1), and soluble receptors IL-1Rα and IL-2Rα.
- Assessment of DNTs in vivo: Peripheral blood aspirates will be analyzed by FACS to assess and quantify the percentages of DNTs before infusion, then 6, 24, 48 hours, 5d, 7d after each DNT infusion, and 14d, 21d, 28d after the 3^rd^ infusion.
- Electrocardiogram (ECG): ECGs should be performed when clinically indicated.
- Bone Marrow Analysis: Bone marrow aspirates will be obtained before the first cycle of DNTs therapy and 28 days after the 3^rd^ infusion. After achieving CR, analyze once a month.
  1. **Follow-up**

Patients will be followed-up for survival data for 2 year from the date of start of treatment. Blood routine, biochemistry and bone marrow studies will be obtained monthly as clinically indicated to assess disease. Analyses will include morphologic examination of the aspirate, FACS, and cytogenetics when indicated.

1. **TREATMENT RESPONSE/OUTCOME ASSESSMENT**
   1. **Main Outcome Measurement**

Cumulative relapse rate at 6 months post-treatment.

- 1. **Secondary Outcome Measurement**

Relapse-free survival (RFS), GvHD-free relapse-free survival (GRFS), and 2-year overall survival (OS) post-cell infusion.

- 1. **Safety Measurement**
- Safety assessment indicators include vital signs, clinical laboratory tests (complete blood count, blood biochemistry, coagulation function, urinalysis), 12-lead electrocardiogram (ECG), and physical examination. Throughout the study, research center staff will monitor adverse events.
- Adverse Events (AEs): The occurrence of all AEs and serious adverse events (SAEs) will be tracked. Changes from baseline in laboratory tests (including complete blood count, urinalysis, blood biochemistry, coagulation function), 12-lead ECG, physical examination, and vital signs (temperature, respiration, blood pressure, and pulse) will be analyzed.
- Dose-limiting toxicities (DLTs) and maximum tolerated dose (MTD) (Phase I only): Cytokine release syndrome (CRS) will be graded according to the ASTCT 2019 criteria, while other AEs will be graded based on the NCI-CTCAE version 5.0. The frequency and severity of DLTs will be assessed to determine the MTD for the subjects.
  1. **Response Assessment**
- Complete Remission (CR): Bone marrow blasts < 5%; absence of circulating blasts and blasts with Auer rods; absence of extramedullary disease; ANC≥1.0×10^9^/L; platelet count ≥100×10^9^/L;
- CR without MRD (CR MRD^-^): If studied pretreatment, CR with negativity for a genetic marker by RT-qPCR, or CR with negativity by MFC;
- CR with incomplete recovery (CRi): All CR criteria except for residual neutropenia (< 1.0 ×10^9^/L or thrombocytopenia (< 100 ×10^9^/L);
- Morphologic leukemia-free state (MLFS): Bone marrow blasts < 5%; absence of blasts with Auer rods; absence of extramedullary disease; no hematologic recovery required;
- Partial Remission (PR): All hematologic criteria of CR; decrease of bone marrow blast percentage to 5% to 25%; and decrease of pretreatment bone marrow blast percentage by at least 50%;
- Treatment failure: Failure to achieve CR or PR
  1. **Relapse**
- Hematologic relapse: after achieving CR MRD^-^, CR, or CRi in AML patients, the reappearance of leukemic cells in peripheral blood or ≥5% blasts in bone marrow (excluding other causes such as bone marrow regeneration after consolidation chemotherapy), or leukemic cell infiltration at extramedullary sites.
- Molecular relapse: after achieving CR MRD^-^, the reappearance of MRD positivity detected by standard methods [e.g., RT-qPCR or multiparametric flow cytometry (MFC) with a sensitivity of at least 10^-3^].
  1. **Survival**
- Overall Survival (OS): From the date of entry into the clinical study until death from any cause.
- Relapse-Free Survival (RFS):  the time from study enrollment until documented disease relapse, or death from any cause.

1. **ADVERSE EVENT REPORTING**
   1. **Definitions**
      1. **Adverse Event**

An AE is any undesirable sign, symptom or medical condition or experience that develops or worsens in severity after starting the first study treatment in the protocol, even if the event is not considered to be related to the study.

- - 1. **Serious Adverse Event**

A SAE is any adverse event, occurring at any dose and regardless of causality that:

- Fatal
- Life-threatening
- Requires or prolongs hospital stay
- Results in persistent or significant disability or affecting the ability to work
- [Congenital](javascript:;) [malformation](javascript:;)
- Carcinogenic
- Important medical event means an event that will not immediately be life-threatening or result in death or hospitalization, but may endanger the subject or require measures to prevent the occurrence of any of the above. For example, angioneurotic edema that do not require tracheal intubation but require corticosteroid therapy, or intensive treatment of bronchospasm.
  1. **Attribution**

Attribution is the relationship between an adverse event or serious adverse event and the study treatment. Attribution will be assigned as follows:

- Definite: The AE is clearly related to the study treatment.
- Probable: The AE is likely related to the study treatment.
- Possible: The AE may be related to the study treatment.
- Unlikely: The AE is doubtfully related to the study treatment.
- Unrelated: The AE is clearly NOT related to the study treatment.
  1. **Procedures for AE and SAE Recording and Reporting**
- The adverse event report form should be filled in truthfully during the study. Record the time, severity, duration, measures and outcome of AE. AE should be recorded in the Adverse event Reporting Form of CRF.
- Any SAE must be reported to the [major](javascript:;) [investigator](javascript:;), the ETHICS committee and [research](javascript:;) [center](javascript:;)  as soon as possible but no later than 24 hours. The investigator must fill out the SAE form, recording the time, severity, duration, measures and outcome of SAE.
  1. **Follow-up of AE**

AE should be followed until remission or stable symptoms, and follow-up of SAE may need to continue until the end of the study.

- 1. **Gestation**

If patient get pregnant during the trial, stop the drug immediately and fill in the pregnancy report form of clinical trial. To ensure the safety of each patient, researcher should report to the sponsor within 24 hours of knowing the pregnancy. Patient should be followed up until the outcome is clear, including abortion or automatic termination of pregnancy, details of delivery, presence of any birth defects, or congenital [malformation](javascript:;), or maternal and neonatal complications.

1. **QUALITY MANAGEMENT**
   1. **Investigators**

- Investigators qualification: Investigators participating in clinical trials must be checked qualification and have the professional background and ability to conduct clinical trials.
- Training of researchers: Receiving training programs arranged by clinical trial institution for each project is a prerequisite to qualify investigators. After the approval of the medical ethics committee, the Principle Investigator (PI) will organize training.
  1. **Clinical Monitors**

Representatives of the Sponsor will conduct a site initiation visit and periodically audit, at mutually convenient times during and after the study, all CRFs and corresponding source documents for each subject. The monitors will verify that investigational products are properly stored and accounted for, verify that subjects’ consent for study participation has been properly obtained and documented, confirm that research subjects entered into the study meet inclusion and exclusion criteria.

- 1. **Standardized Operation**

The sponsor complies with the Drug Clinical Trial Quality Management Standard (2003 edition) and the Drug Registration Regulations. DNTs will be expanded ex vivo under GMP condition. Ensure the high quality for them.

- 1. **Laboratory Examination**

The research center will conduct laboratory tests in accordance with standard operating procedures (SOP).

1. **DATA HANDLING AND RECORD KEEPING**
   1. **Data Collection**

The Case Report Form (CRF) will comprise a set of forms capturing details of eligibility, baseline characteristics, treatment and outcome details etc. All entries will be entered into an electronic data capture system (EDC). PI is responsible for assuring that the data entered into eCRF is complete, accurate, and that entry and updates are performed in a timely manner.

- 1. **Data Retention**
- It is the investigator’s responsibility to retain study original documents. Original data include original records, informed consent signed by subjects, laboratory data, imaging data, ECG, medication records, etc.
- The researchers center should keep the basic data of subjects for a longer time according to the regulations (at least 5 years).

1. **SAMPLE SIZE**

Part A is a dose escalation trial with two dose groups, 6-12 patients planned to be enrolled. Part B is a dose-expansion randomized controlled trial in which 40-60 patients will receive RC1012 infusions at RP2D dose levels.

1. **REFERENCE**

1. Zeiser R, Beelen DW, Bethge W, Bornhäuser M, Bug G, Burchert A, et al. Biology-Driven Approaches to Prevent and Treat Relapse of Myeloid Neoplasia after Allogeneic Hematopoietic Stem Cell Transplantation. Biol Blood Marrow Transplant. 2019;25(4):e128-e40.

2. Webster JA, Luznik L, Gojo I. Treatment of AML Relapse After Allo-HCT. Front Oncol. 2021;11:812207.

3. Thol F, Schlenk RF, Heuser M, Ganser A. How I treat refractory and early relapsed acute myeloid leukemia. Blood. 2015;126(3):319-27.

4. Zhang ZX, Yang L, Young KJ, DuTemple B, Zhang L. Identification of a previously unknown antigen-specific regulatory T cell and its mechanism of suppression. Nat Med. 2000;6(7):782-9.

5. Young KJ, DuTemple B, Phillips MJ, Zhang L. Inhibition of graft-versus-host disease by double-negative regulatory T cells. J Immunol. 2003;171(1):134-41.

6. Merims S, Li X, Joe B, Dokouhaki P, Han M, Childs RW, et al. Anti-leukemia effect of ex vivo expanded DNT cells from AML patients: a potential novel autologous T-cell adoptive immunotherapy. Leukemia. 2011;25(9):1415-22.

7. He KM, Ma Y, Wang S, Min WP, Zhong R, Jevnikar A, Zhang ZX. Donor double-negative Treg promote allogeneic mixed chimerism and tolerance. Eur J Immunol. 2007;37(12):3455-66.

8. Pan T, Ding P, Huang A, Tang B, Song K, Sun G, et al. Reconstitution of double-negative T cells after cord blood transplantation and its predictive value for acute graft-versus-host disease. Chin Med J (Engl). 2024;137(10):1207-17.

1. **APPENDIX 1 – [Common Terminology Criteria for Adverse Events (CTCAE) v5.0](https://ctep.cancer.gov/protocolDevelopment/electronic_applications/ctc.htm#ctc_50)**

| Grade | Symptoms |
| --- | --- |
| 1 | Mild: Asymptomatic or mild symptoms that require only minimal intervention. The condition is self-limiting or easily manageable. |
| 2 | Moderate symptoms requiring some medical intervention or medication but not immediately life-threatening. Activities of daily living (ADLs) may be mildly restricted. |
| 3 | Severe symptoms requiring hospitalization or significant medical intervention. There’s a considerable limitation on ADLs. |
| 4 | Life-threatening consequences that require urgent intervention. |
| 5 | Death related to the adverse event. |

**APPENDIX 2 – EOCG Status**

| **ECOG Status** | **Description** |
| --- | --- |
| 0 | Asymptomatic, fully active and able to carry on all predisease performance without restrictions. |
| 1 | Symptomatic, fully ambulatory but restricted in physically strenuous activity and able to carry out performance of a light or sedentary nature, e.g., light housework, office work. |
| 2 | Symptomatic, ambulatory and capable of all self-care but unable to carry out any work activities. Up and about more than 50% of waking hours: in bed less than 50% of day. |
| 3 | Symptomatic, capable of only limited self-care, confined to bed or chair more than 50% of waking hours but not bedridden. |
| 4 | Completely disabled. Cannot carry on any self-care. Totally bedridden. |
| 5 | Dead |
