# Supplementary material for: Prophylactic infusion of allogeneic double-negative T cells as immune modulators to prevent relapse in high-risk AML patients post-Allo-HSCT: a phase I trial
Source: Exp Hematol Oncol. 2025 Jul 2;14:90. doi: 10.1186/s40164-025-00680-1 (PMC12224462; doi:10.1186/s40164-025-00680-1)
Supplement: Supplementary file 2 — Supplementary Material 2 [file 40164_2025_680_MOESM2_ESM.docx]

**Supplementary Data**

**Methods**

***Study design***

This single-center, prospective, open-label clinical trial was to evaluate the safety and efficacy of prophylactic allogeneic double-negative T (allo-DNT) cell infusion in acute myeloid leukemia (AML) patients after allogeneic hematopoietic stem cell transplantation (allo-HSCT). The study incorporated a dose-escalation component to determine the maximum tolerated dose (MTD) of allo-DNT cells.​ MTD defined as the highest dose level at which ≤1 of 6 patients experienced a DLT.

Eligible participants were assigned to two sequential dose cohorts:​ Cohort 1: Patients 1–3 received allo-DNT infusions at a dose of 1 × 10⁸ cells/kg.​ Cohort 2: Patients 4–6 received allo-DNT infusions at an escalated dose of 1.5 × 10⁸ cells/kg.

Eligible participants received planned three doses of allo-DNT cells from a third-party donor, with a 28-day interval within 60 to 100 days post-transplantation. Following each infusion, patients were monitored for adverse reactions over a 28-day period, with subsequent monthly follow-ups for up to two years. The primary aim was to assess the occurrence of adverse events and dose-limiting toxicity (DLT). Secondary objectives were to analyze the pharmacokinetics of allo-DNT cells and evaluate the six-month cumulative incidence of relapse (CIR), overall survival (OS) and progression-free survival (PFS). Adverse events were graded and reported according to the National Cancer Institute's Common Terminology Criteria for Adverse Events (CTCAE), version 5.0.

***Patients and healthy donor samples***

Eligible participants were aged 18 to 70 years, diagnosed with AML according to the 2016 World Health Organization (WHO) criteria, and had received allo-HSCT within 60 to 100 days prior to enrollment. Post-transplantation, patients exhibited less than 5% malignant primitive cells in the bone marrow, and short tandem repeat polymerase chain reaction (STR-PCR) confirmed complete donor chimerism.

To be considered as high-risk for relapse after allo-HSCT, patients must meet at least one of the following criteria:

1. Failure to achieve remission after two courses of induction chemotherapy.
2. Prior history of myelodysplastic syndrome (MDS) or myeloproliferative neoplasm (MPN).
3. High leukocyte count (≥100×10^9/L) combined with central nervous system leukemia (CNSL).
4. Positive minimal residual disease (MRD) before HSCT.
5. Non-remission or disease progression prior to HSCT.
6. Presence of high-risk cytogenetic factors (excluding those treatable with targeted therapies).

For the manufacturing of DNT cells, peripheral blood was collected from pre-screened healthy donors and transported to the Wyze Biotech GMP facility. The cells were cultured ex vivo for 14–17 days, then harvested, cryopreserved, and stored for future use. To enable tracking of infused allogeneic DNT cells in patients, HLA-A2 mismatched donors were selected for treatment.

The purity of CD4/CD8 negative T cells was more than 85% of CD3⁺ T cells before and after expansion. The cells were cryopreserved in liquid nitrogen before use. The trial received approval from the Ethics Committee of the First Affiliated Hospital of the University of Science and Technology of China. Informed consent was obtained from all patients and donors in accordance with the Declaration of Helsinki.

***Efficacy evaluation***

Disease burden was assessed via bone marrow biopsy. Levels of DNTs in peripheral blood and bone marrow were quantified using a BD LSRFortessa™ flow cytometer, and data analysis was performed with FlowJo™ software. To enable tracking of infused allogeneic DNT cells in patients, HLA-A2 mismatched donors were selected for treatment. Donor-derived DNTs were identified using flow cytometry by detecting HLA-A2 expression on CD3⁺CD4⁻CD8⁻CD56⁻ lymphocytes. The absolute count of DNTs was calculated by multiplying their percentage (CD3⁺CD4⁻CD8⁻CD56⁻) of total lymphocytes. Cytokine levels were measured using a Cytokine Combination Assay Kit according to the manufacturer's instructions (cell-genebio). Briefly, plasma samples were collected from patients at designated time points post-infusion. The assay was performed using a multiplex bead-based immunoassay, which allows for the simultaneous quantification of multiple cytokines, including IFN-γ, TNF-α, IL-2, IL-4, IL-6 and IL-10. Samples were processed following the kit protocol, and fluorescence intensities were detected using a flow-based analyzer. Cytokine concentrations were calculated using a standard curve generated from known concentrations of recombinant cytokines. Data were analyzed using the accompanying software.

***Co-culture experiment***

DNTs were expanded ex vivo following the protocol established by Zhang et al. In brief, peripheral blood mononuclear cells (PBMCs) were isolated from healthy donors, and DNTs were enriched by depleting CD4⁺ and CD8⁺ T cells using a CD4 and CD8 depletion cocktail (Stemcell Technologies). The enriched DNTs were cultured in AIM V medium supplemented with 10% fetal bovine serum (FBS) and 250 IU/mL interleukin-2 (IL-2; Proleukin, Novartis Pharmaceuticals) on plates coated with anti-CD3 antibody (OKT3; 5 μg/mL) for 3 days. Subsequently, the cells were transferred to complete medium containing AIM V, 250 IU/mL IL-2, and 0.1 μg/mL OKT3, and cultured for an additional 11 days. On day 14, the cells were harvested, and DNT cell purity (CD3⁺CD4⁻CD8⁻CD56⁻) was assessed via flow cytometry. To evaluate the effect of allogeneic DNT cells on CD8⁺ T cells, DNTs from donor A were co-cultured with CD8⁺ T cells from donor B of AML patients at a 4:1 ratio in AIM V medium supplemented with 100 IU/mL IL-2 for 3–4 days. After the co-culture, CD8⁺ T cells were analyzed by flow cytometry for interferon-gamma (IFN-γ) and granzyme (GZM) B expression.

***Microarrays for cytokine release***

To assess cytokine production, supernatants from co-cultures of allo-DNTs with CD8⁺ T cells (experimental group), as well as from CD8⁺ T cells cultured alone (control group), were collected. The Human Cytokine Array C5 (RayBiotech, AAH-CYT-5) was employed to detect 80 different growth factors and inflammatory cytokines (Additional file 1, supplementary table 4) following the manufacturer's instructions. Differential expression of cytokines between the experimental and control groups was analyzed using Student's t-test with continuity correction (adjusted p < 0.05). Cytokines exhibiting a fold change greater than 1.5 and an adjusted p-value less than 0.05 were considered significantly different. These differentially expressed cytokines were further subjected to functional enrichment analysis using Gene Ontology (GO) terms and Kyoto Encyclopedia of Genes and Genomes (KEGG) pathways via the Metascape platform.

***Statistics***

The clinical evaluation cutoff date was February 1, 2025. Patient characteristics, safety profiles, and follow-up data were tabulated and summarized using descriptive statistics. Kaplan-Meier curves illustrated OS and PFS, with both metrics calculated from HSCT, designated as "day 0." Cumulative incidence curves were built in a competing risk setting, with death considered a competing event, to calculate the cumulative incidences of relapse. Data analyses were conducted using IBM SPSS Statistics software (version 23.0; IBM Corp., Armonk, NY, USA) and EZR version 1.61 (Microsoft Corporation, USA). All figures were generated using GraphPad Prism 9 software (GraphPad Software, San Diego, CA, USA). A p-value of less than 0.05 was deemed statistically significant. Significance levels were indicated as follows: *p < 0.05; **p < 0.01; ***p < 0.001.

**Supplementary figure**

Supplementary Figure 1. Cytokines level after infusion of allo-DNTs between recurrent and non-recurrent patients. Measurement of plasma (A) IFN-γ , (B) TNF-α, (C) IL-2, (D) IL-4 and (E) IL-10 at different time points (day 0, 1, 2, 3, 4, 7, 14, 21, 28) during three cycles of DNT infusion.


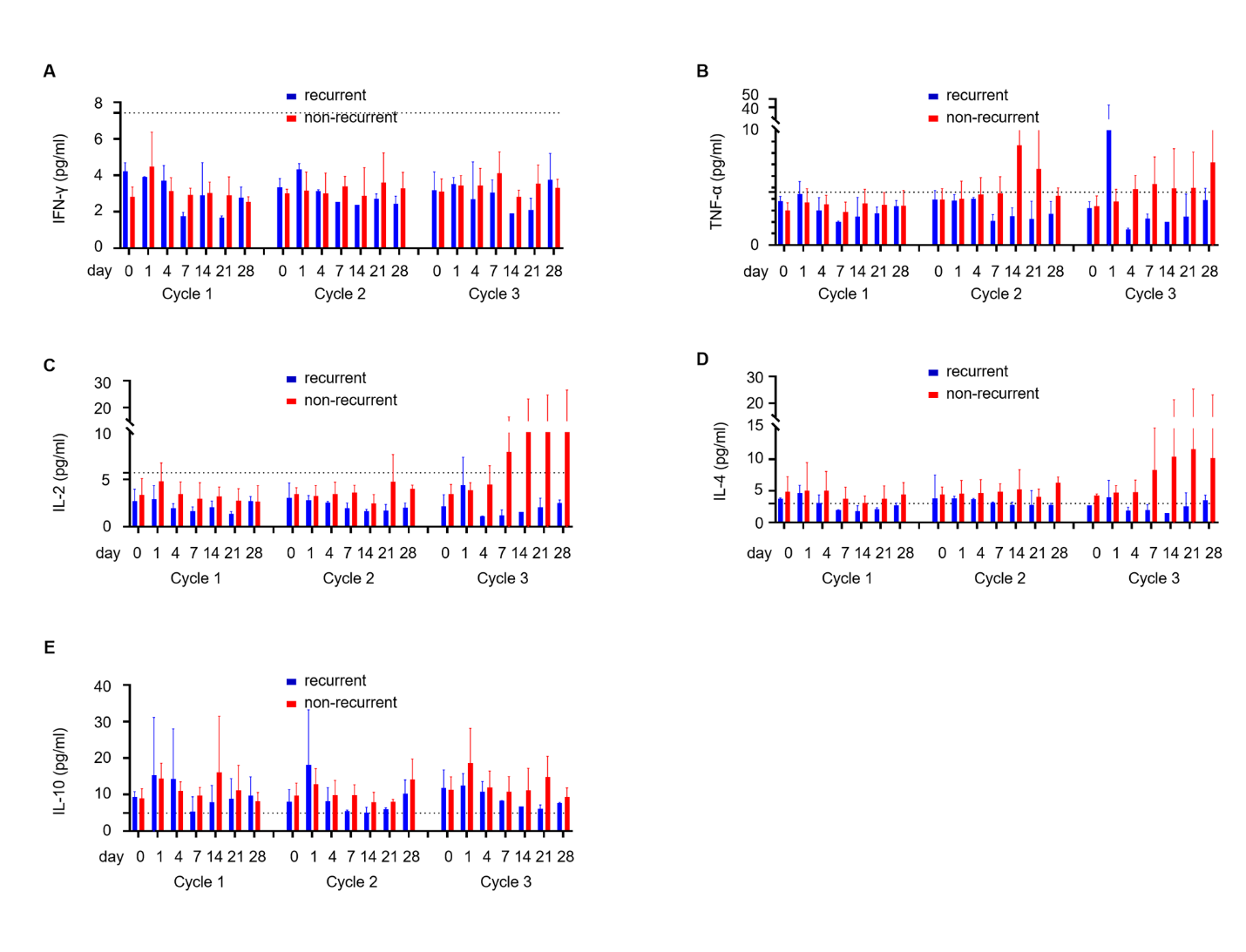


Supplementary Figure 2. Immunophenotype and cytotoxic markers of recipients’ immune cells. Immunophenotype of CD4⁺ T cells (A), CD8⁺ T cells (B) and DNTs (C) of each patient at different time points (day 0, 1, 2, 3, 4, 7, 14, 21, 28) during three cycles of DNT infusion. n=6, Data are represented as mean ± SEM. (D-F) Total absolute number of GZMA⁺, GZMB⁺, GZMK⁺ DNTs. (G-H) Total absolute number of GZMA⁺ and GZMK⁺ CD8⁺ T cells. (I-J) Total absolute number of GZMA⁺ and GZMK⁺ CD8⁺ T. (effector memory [EM]: CD62L−, CD45RA−; central memory [CM]: CD62L+, CD45RA−; GZM: granzyme).


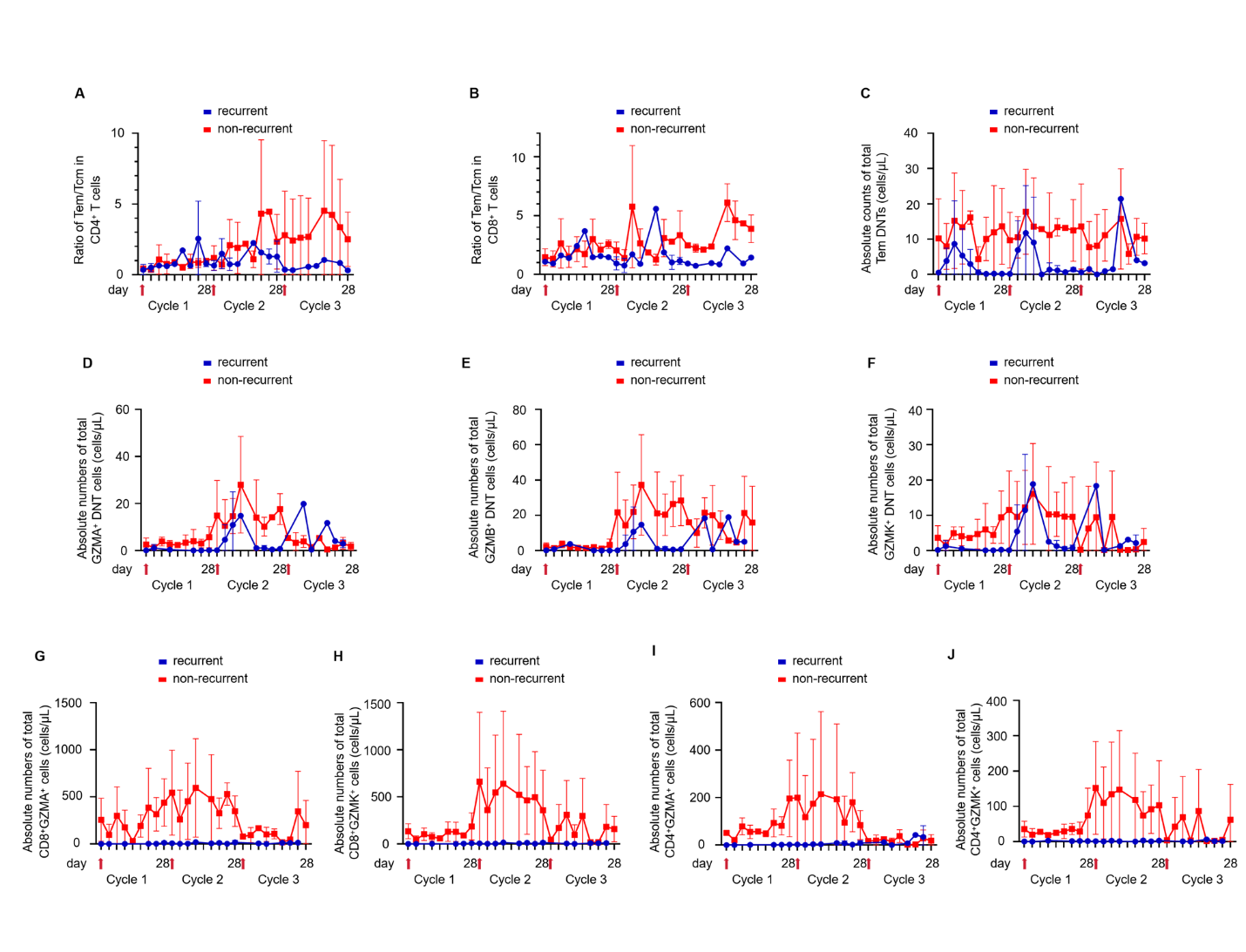


Supplementary Figure 3. DNTs activate AML-CD8⁺ T cells. CD8⁺ T cells from AML patients (n=4) co-cultured with allogeneic DNTs were used as the experimental group (AML-CD8 co-cultured with allo-DNTs), and CD8⁺ T cells cultured alone were used as the control group (AML-CD8). Following a 3-4 days co-culture, flow cytometry was performed to measure IFN-γ (A) and granzyme B (B) levels. The supernatants from the co-culture were subjected to cytokine release detection. Cytokine profiles of significantly upregulated (red) and downregulated (green) cytokines (p < 0.01, fold change＞1.5) are displayed in heatmap (C); KEGG and GO enrichment analyses of the differentially expressed factors are presented in (D). The size of each bubble represents the number of differentially cytokines enriched in the corresponding pathway. n = 3 patients/group. Paired student’s t-test were used. *p < 0.05; **p < 0.01; ***p < 0.001; ****p < 0.0001.


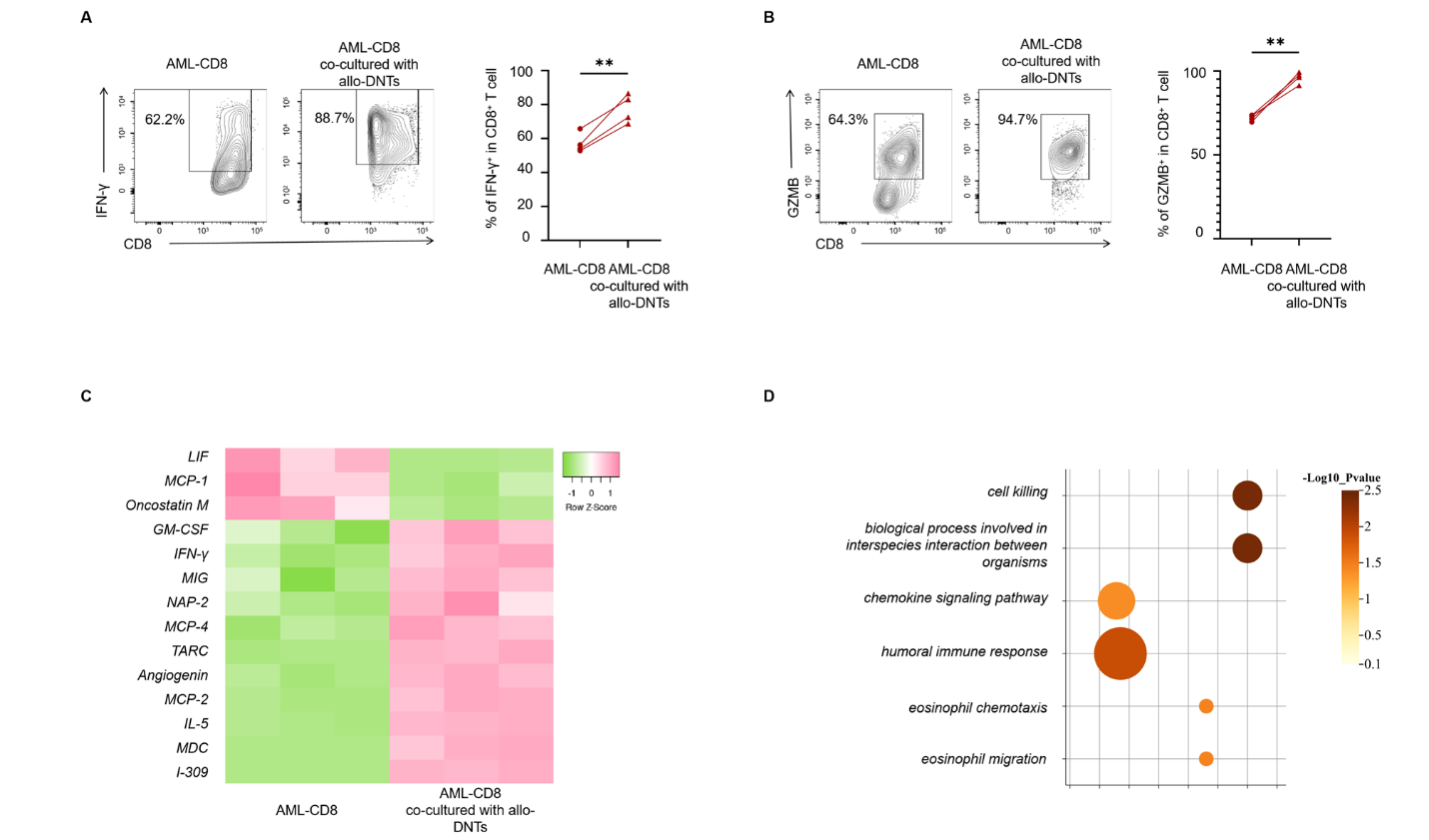


**Supplementary table：**

Supplementary table 1. Adverse events observed in the 6 study patients

| Adverse Event | Grade 1 or 2 | Grade 3 | Grade 4 |
| --- | --- | --- | --- |
|  | no. of patients | | |
| **Cytokine release syndrome** | 1# | 0 | 0 |
| **Infusion reactions** | 5# | 0 | 0 |
| **Acute graft-versus-host disease** | 1 | 0 | 0 |
| Gastrointestinal GVHD | 1 | 0 | 0 |
| **Chronic graft-versus-host disease** | 1 | 0 | 0 |
| **Immune-related adverse events** |  |  |  |
| Chills | 0 | 0 | 0 |
| Decrease in blood pressure | 0 | 0 | 0 |
| toxic erythema | 1# | 0 | 0 |
| Shortness of breath | 0 | 0 | 0 |
| Pneumonitis | 2 | 0 | 0 |
| Colitis | 0 | 0 | 0 |
| **Nervous system disorders** |  |  |  |
| Headache | 0 | 0 | 0 |
| Dizziness | 0 | 0 | 0 |
| Encephalopathy | 0 | 0 | 0 |
| Delirium | 0 | 0 | 0 |
| Tremor | 0 | 0 | 0 |
| Cognitive disturbance | 0 | 0 | 0 |
| Confusion | 0 | 0 | 0 |
| Involuntary movements | 0 | 0 | 0 |
| Memory impairment | 0 | 0 | 0 |
| **Blood and lymphatic system disorders** |  |  |  |
| Neutropenia | 0 | 0 | 0 |
| Lymphopenia | 0 | 0 | 0 |
| Thrombocytopenia | 0 | 0 | 0 |
| Anemia | 0 | 0 | 0 |
| **Cardiac disorders** |  |  |  |
| Chest pain | 0 | 0 | 0 |
| Hypertension | 1 | 0 | 0 |
| Atrial fibrillation | 0 | 0 | 0 |
| Tachycardia | 0 | 0 | 0 |
| **Gastrointestinal disorders** |  |  |  |
| Nausea | 0 | 0 | 0 |
| Vomit | 0 | 0 | 0 |
| Abdominal pain | 0 | 0 | 0 |
| Diarrhea | 1 | 0 | 0 |
| Colonic hemorrhage | 0 | 0 | 0 |
| Elevated ALT/AST | 1 | 0 | 0 |
| **Constitutional event (General disorders)** |  |  |  |
| Fatigue | 0 | 0 | 0 |
| Insomnia | 0 | 0 | 0 |
| **Infection event** |  |  |  |
| Bacteria infection | 4 | 0 | 0 |
| Viral infection | 3 | 2 | 0 |
| CMV | 3 | 2 | 0 |
| EBV | 0 | 0 | 0 |
| Fungus infection | 0 | 0 | 0 |
| **Others** |  |  |  |
| Blurred vision | 0 | 0 | 0 |
| Pleural effusion | 0 | 0 | 0 |
| Skin discoloration | 0 | 0 | 0 |
| Joint pain | 1 | 0 | 0 |
| Limb edema | 0 | 0 | 0 |
| **Investigations (Laboratory values)** |  |  |  |
| Electrolyte imbalance | 4 | 0 | 0 |
| Hyperlipemia | 3 | 0 | 0 |
| Hypoalbuminemia | 2 | 0 | 0 |
| Hypoglobulinemia | 1 | 0 | 0 |
| Ferrugination | 3 | 0 | 0 |
| Lower fibrinogen | 1 | 0 | 0 |
| hematuresis | 1 | 0 | 0 |

Listed are all adverse events that were observed in the 6 patients from the time of DNT cell infusion until 30 days after last infusion, regardless of whether the investigators attributed the events to the treatment. Abnormalities caused by the original disease are not listed. ALT, Alanine aminotransferase; AST, Aspartate aminotransferase.

# DNT-related toxicity

Supplementary table 2. Patient treatment after DNT cell infusions

| Pt No. | Salvage/maintenance therapy after DNT |
| --- | --- |
| 01002 | Clinical trial |
| 01003 | None |
| 01005 | None |
| 01007 | IFN-α2β x4  IFN-α2β + AZA |
| 01008 | None |
| 01009 | None |

Abbreviations: IFN-α2β, interferon-α2β; AZA, azacitidine

Supplementary table 3. PK of DNT cells after infusion

| Donors’ DNT cells (counts/ml) | | | | | | | | | | |
| --- | --- | --- | --- | --- | --- | --- | --- | --- | --- | --- |
| Dose level | 1×10^8^/kg | | | | | 1.5×10^8^/kg | | | | |
|  | 01002 | 01003 | 01005 | Average | SD | 01007 | 01008 | 01009 | Average | SD |
| Before infusion | 0 | 0 | 0 | 0 | 0 | 0 | 0 | 0 | 0 | 0 |
| C1D1-3h | 2913 | 9291 | 2907 | 5037 | 3684 | 7322 | 7549 | 24302 | 13057 | 9738 |
| C1D2 | 3904 | 1884 | 6231 | 4006 | 2175 | 22573 | 6287 | 45417 | 24759 | 19656 |
| C1D3 | 3463 | 496 | / | 1980 | 2098 | 13192 | / | 37728 | 25460 | 17350 |
| C1D4 | 1959 | 0 | 3342 | 1767 | 1679 | 7013 | 1540 | 19855 | 9469 | 9401 |
| C1D8 | 671 | 0 | 44 | 238 | 375 | 1326 | 233 | 904 | 821 | 551 |
| C1D15 | 289 | 0 | 41 | 110 | 156 | 228 | 0 | 108 | 112 | 114 |
| C1D22 | 340 | 0 | 0 | 113 | 196 | 89 | 111 | 242 | 147 | 82 |
| C1D29 | 963 | 0 | 74 | 346 | 536 | 116 | 163 | 103 | 127 | 32 |
| C2D1  Before infusion | 777 | 0 | 0 | 259 | 448 | 0 | 0 | 162 | 54 | 94 |
| C2D1-3h | 2497 | 5559 | 659 | 2905 | 2475 | 14466 | 6186 | 20918 | 13857 | 7385 |
| C2D2 | 7587 | 605 | 535 | 2909 | 4052 | 26577 | 2266 | 36533 | 21792 | 17627 |
| C2D3 | 32505 | / | / | 32505 | / | / | / | / | / | / |
| C2D4 | 10160 | 130 | 289 | 3526 | 5745 | 24377 | 380 | 76759 | 33839 | 39059 |
| C2D8 | 1319 | 0 | 0 | 440 | 762 | 3028 | 153 | 182 | 1121 | 1651 |
| C2D15 | 1041 | 0 | 0 | 347 | 601 | 2259 | 74 | 432 | 921 | 1172 |
| C2D22 | 2085 | 0 | 0 | 695 | 1204 | 973 | 215 | 277 | 488 | 421 |
| C2D29 | 843 | 0 | 0 | 281 | 487 | 853 | 98 | 0 | 317 | 467 |
| C3D1  Before infusion | 67 | 0 | 0 | 22 | 39 | 841 | 0 | 87 | 309 | 462 |
| C3D1-3h | 2022 | 5833 | 1079 | 2978 | 2517 | 14779 | 5201 | 15252 | 11744 | 5672 |
| C3D2 | 543 | 990 | 238 | 591 | 378 | 30247 | 431 | 19377 | 16685 | 15089 |
| C3D3 | 90 | / | / | 90 | / | / | / | / | / | / |
| C3D4 | 112 | 92 | 124 | 109 | 16 | 29235 | 243 | 5009 | 11496 | 15546 |
| C3D8 | 494 | 0 | 0 | 165 | 285 | 5925 | 0 | 93 | 2006 | 3394 |
| C3D15 | N/D | 0 | 0 | 0 | 0 | 4655 | 284 | / | 2469 | 3091 |
| C3D22 | 89 | 0 | 0 | 30 | 51 | 4001 | 0 | / | 2001 | 2829 |
| C3D29 | 0 | 0 | 0 | 0 | 0 | 43 | 0 | 0 | 14 | 25 |
| D120 | / | 0 | 0 | 0 | / | / | 136 | 0 | 68 | 96 |
| D150 | 0 | 0 | 0 | 0 | / | / | 0 | 164 | 82 | 116 |
| D180 | / | 0 | 0 | 0 | / | / | 0 | 0 | 0 | 0 |
| D270 | / | 0 | 0 | 0 | / | / | / | / | / | / |
| D360 | / | 0 | 347 | / | / | / | 375 | 0 | / | / |

| Recipients’ DNT cells (counts/ml) | | | | | | | | | | |
| --- | --- | --- | --- | --- | --- | --- | --- | --- | --- | --- |
| Dose level | 1×10^8^/kg | | | | | 1.5×10^8^/kg | | | | |
|  | 01002 | 01003 | 01005 | Average | SD | 01007 | 01008 | 01009 | Average | SD |
| Before infusion | 4550 | 26433 | 44604 | 25195 | 20056 | 111 | 2285 | 6472 | 2956 | 3233 |
| C1D1-3h | 830 | 11000 | 3852 | 5227 | 5223 | 55 | 2831 | 3125 | 2004 | 1694 |
| C1D2 | / | / | / | / | / | / | / | / | / | / |
| C1D3 | / | / | / | / | / | / | / | / | / | / |
| C1D4 | 406 | 23241 | 28909 | 17519 | 15088 | 62 | 3157 | 5994 | 3071 | 2967 |
| C1D8 | 286 | 19623 | 25789 | 15233 | 13306 | 117 | 1633 | 11059 | 4270 | 5928 |
| C1D15 | 1156 | 65499 | 30301 | 32319 | 32219 | 127 | 5929 | 20559 | 8872 | 10529 |
| C1D22 | 1214 | 124682 | 26491 | 50796 | 65224 | 134 | 3094 | 20597 | 7942 | 11059 |
| C1D29 | 963 | 67675 | 34991 | 34543 | 33358 | 139 | 6436 | 30096 | 12224 | 15795 |
| C2D1  Before infusion | 388 | 54257 | 19178 | 24608 | 27342 | 111 | 11931 | 15678 | 9240 | 8125 |
| C2D1-3h | 624 | 32705 | 8288 | 13873 | 16754 | 46 | 5322 | 10552 | 5307 | 5253 |
| C2D2 | / | / | / | / | / | / | / | / | / | / |
| C2D3 | / | / | / | / | / | / | / | / | / | / |
| C2D4 | 1016 | 53866 | 17422 | 24101 | 27051 | 275 | 14808 | 25381 | 13488 | 12605 |
| C2D8 | 552 | 63987 | 16233 | 26924 | 33041 | 444 | 11168 | 19099 | 10237 | 9362 |
| C2D15 | 558 | 38392 | 26025 | 21658 | 19291 | 414 | 19140 | 26011 | 15188 | 13248 |
| C2D22 | 847 | 40578 | 28229 | 23218 | 20334 | 216 | 42528 | 28468 | 23737 | 21549 |
| C2D29 | 6838 | 43810 | 18232 | 22960 | 18934 | 244 | 27506 | 22388 | 16713 | 14490 |
| C3D1  Before infusion | 3390 | 55272 | 16443 | 25035 | 26987 | 440 | 19015 | 25338 | 14931 | 12942 |
| C3D1-3h | 2065 | 16991 | 7681 | 8912 | 7539 | 190 | 19384 | 13919 | 11164 | 9889 |
| C3D2 | / | / | / | / | / | / | / | / | / | / |
| C3D3 | / | / | / | / | / | / | / | / | / | / |
| C3D4 | 4321 | 39727 | 19341 | 21130 | 17771 | 1058 | 18585 | 26136 | 15259 | 12865 |
| C3D8 | 45688 | 38497 | 15808 | 33331 | 15596 | 1296 | 13924 | 27400 | 14207 | 13054 |
| C3D15 | N/D | 32008 | 15183 | 23595 | 11897 | 3411 | 16545 | / | 9978 | 9287 |
| C3D22 | 9695 | 54543 | 16843 | 27027 | 24096 | 2751 | 29399 | / | 16075 | 18843 |
| C3D29 | 8516 | 35336 | 16350 | 20067 | 13791 | 859 | 6961 | 27436 | 11752 | 13921 |
| D120 | / | 122705 | 20130 | 71418 | / | / | 21223 | 46011 | 33617 | 17528 |
| D150 | 50399 | 169211 | 19098 | 79569 | / | / | 13879 | 27381 | 20630 | 9548 |
| D180 | / | 189343 | 17510 | 103427 | / | / | 6660 | 30990 | 18825 | 17204 |
| D270 | / | 124848 | 33522 | 79185 | / | / | / | / | / | / |
| D360 | / | 41761 | 107275 | 74518 | / | / | 18389 | 25040 | 21715 |  |

| Total DNT cells (counts/ml) | | | | | | | | | | |
| --- | --- | --- | --- | --- | --- | --- | --- | --- | --- | --- |
| Dose level | 1×10^8^/kg | | | | | 1.5×10^8^/kg | | | | |
|  | 01002 | 01003 | 01005 | Average | SD | 01007 | 01008 | 01009 | Average | SD |
| C1D1  Before infusion | 4550 | 26433 | 44604 | 25195 | 20056 | 111 | 2285 | 6472 | 2956 | 3233 |
| C1D1-3h | 3743 | 20291 | 6759 | 10264 | 8813 | 7377 | 10380 | 27427 | 15061 | 10814 |
| C1D2 | 4988 | 19826 | 25554 | 16789 | 10614 | 22754 | 10742 | 52694 | 28730 | 21605 |
| C1D3 | 3958 | 20552 | / | 12255 | 11734 | 13311 | / | 47127 | 30219 | 23912 |
| C1D4 | 2365 | 23241 | 32251 | 19286 | 15331 | 7075 | 4698 | 25849 | 12540 | 11587 |
| C1D8 | 957 | 19623 | 25833 | 15471 | 12947 | 1443 | 1867 | 11963 | 5091 | 5955 |
| C1D15 | 1445 | 65499 | 30341 | 32428 | 32078 | 355 | 5929 | 20667 | 8983 | 10495 |
| C1D22 | 1554 | 124682 | 26491 | 50909 | 65095 | 223 | 3205 | 20839 | 8089 | 11142 |
| C1D29 | 1926 | 67675 | 35066 | 34889 | 32875 | 255 | 6599 | 30199 | 12351 | 15779 |
| C2D1  Before infusion | 1165 | 54257 | 19178 | 24867 | 26999 | 111 | 11931 | 15841 | 9294 | 8189 |
| C2D1-3h | 3121 | 38264 | 8947 | 16777 | 18835 | 14511 | 11509 | 31470 | 19163 | 10763 |
| C2D2 | 8005 | 52654 | 13487 | 24715 | 24350 | 26689 | 19785 | 48730 | 31735 | 15118 |
| C2D3 | 35354 | 86688 | / | 61021 | 36299 | / | / | / | / | / |
| C2D4 | 11176 | 53997 | 17711 | 27628 | 23069 | 24652 | 15188 | 102140 | 47326 | 47705 |
| C2D8 | 1870 | 63987 | 16233 | 27363 | 32520 | 3472 | 11321 | 19282 | 11358 | 7905 |
| C2D15 | 1599 | 38392 | 26025 | 22005 | 18723 | 2673 | 19214 | 26443 | 16110 | 12185 |
| C2D22 | 2932 | 40578 | 28229 | 23913 | 19191 | 1189 | 42742 | 28746 | 24226 | 21142 |
| C2D29 | 7682 | 43810 | 18232 | 23241 | 18578 | 1096 | 27604 | 22388 | 17030 | 14043 |
| C3D1  Before infusion | 3457 | 55272 | 16443 | 25058 | 26960 | 1280 | 19015 | 25425 | 15240 | 12507 |
| C3D1-3h | 4087 | 22823 | 8759 | 11890 | 9752 | 14969 | 24585 | 29171 | 22908 | 7248 |
| C3D2 | 4755 | 39022 | 9744 | 17840 | 18513 | 30621 | 27816 | 46145 | 34860 | 9873 |
| C3D3 | 2956 | / | / | 2956 |  | / | / | / | / | / |
| C3D4 | 4433 | 39819 | 19465 | 21239 | 17760 | 30293 | 18828 | 31144 | 26755 | 6878 |
| C3D8 | 46182 | 38497 | 15808 | 33495 | 15793 | 7221 | 13924 | 27492 | 16212 | 10328 |
| C3D15 | N/D | 32008 | 15183 | 23595 | 11897 | 8066 | 16829 | / | 12447 | 6196 |
| C3D22 | 9784 | 54543 | 16843 | 27057 | 24064 | 6752 | 29399 | / | 18075 | 16014 |
| C3D29 | 8516 | 35336 | 16350 | 20067 | 13791 | 902 | 6961 | 27436 | 11766 | 13904 |
| D120 | / | 122705 | 20130 | 71418 | / | / | 21359 | 46011 | 33685 | 17431 |
| D150 | 50399 | 169211 | 19098 | 79569 | / | / | 13879 | 27545 | 20712 | 9664 |
| D180 | / | 189343 | 17510 | 103427 | / | / | 6660 | 30990 | / | / |
| D270 | / | 124848 | 33522 | 79185 | / | / | / | / | / | / |
| D360 | / | 41761 | 107621 | 74691 | / | / | 18764 | 25040 | 21902 | / |

Supplementary table 4. Cytokine measurement.

| proteinID | Cocultured CD8 (1) | Cocultured CD8 (2) | Cocultured CD8 (3) | CD8 (1) | CD8 (2) | CD8 (3) | adj.P.Val | foldchange |
| --- | --- | --- | --- | --- | --- | --- | --- | --- |
| I-309 | 11784 | 11706 | 12281 | 1192 | 1202 | 1221 | 3.1E-09 | 9.89 |
| MDC | 16842 | 19680 | 20121 | 2988 | 2645 | 2688 | 9.8E-09 | 6.79 |
| IL-5 | 45916 | 47339 | 48537 | 10131 | 8819 | 8306 | 2.6E-08 | 5.22 |
| TARC | 8824 | 8615 | 9162 | 3181 | 3392 | 3410 | 1.4E-07 | 2.66 |
| MCP-2 | 3714 | 4111 | 4057 | 1233 | 1070 | 1074 | 1.4E-07 | 3.52 |
| LIF | 2232 | 2219 | 2294 | 7607 | 5758 | 6734 | 1.2E-05 | 0.34 |
| Angiogenin | 6484 | 6908 | 6373 | 2371 | 1845 | 2074 | 4.1E-05 | 3.16 |
| IFN- g | 7776 | 8426 | 8586 | 5227 | 4481 | 4697 | 8.0E-05 | 1.72 |
| Oncostatin M | 4399 | 4078 | 4306 | 7459 | 7245 | 6018 | 4.6E-04 | 0.62 |
| GM-CSF | 4750 | 5216 | 4774 | 3666 | 3205 | 2737 | 2.1E-03 | 1.54 |
| MIG | 5475 | 5830 | 5347 | 3618 | 2200 | 2983 | 2.1E-03 | 1.93 |
| MCP-4 | 3405 | 3105 | 3017 | 1316 | 1624 | 1544 | 3.7E-03 | 2.13 |
| NAP-2 | 6218 | 6887 | 5161 | 3484 | 2872 | 2688 | 3.7E-03 | 2.02 |
| MCP-1 | 1080 | 1001 | 1260 | 2537 | 1971 | 1967 | 4.0E-03 | 0.52 |
| MCSF | 3360 | 3204 | 3617 | 2761 | 2106 | 2547 | 8.2E-03 | 1.38 |
| TNF-b | 1193 | 956 | 1117 | 1348 | 1667 | 1345 | 2.9E-02 | 0.75 |
| GRO | 1785 | 2253 | 1822 | 2955 | 2284 | 2746 | 5.4E-02 | 0.73 |
| GCSF | 502 | 308 | 448 | 458 | 1829 | 660 | 1.3E-01 | 0.50 |
| IL-2 | 765 | 640 | 468 | 847 | 744 | 903 | 1.3E-01 | 0.74 |
| HGF | 1754 | 1762 | 1524 | 1476 | 1382 | 1149 | 1.3E-01 | 1.26 |
| MCP-3 | 721 | 678 | 913 | 602 | 523 | 531 | 1.8E-01 | 1.39 |
| Leptin | 1277 | 1304 | 1490 | 1825 | 2062 | 1628 | 2.4E-01 | 0.74 |
| EGF | 3269 | 3949 | 3871 | 3277 | 3139 | 2974 | 2.5E-01 | 1.18 |
| Thrombopoietin | 1244 | 1378 | 1745 | 914 | 1452 | 869 | 2.5E-01 | 1.37 |
| PARC | 2438 | 2265 | 2227 | 3031 | 2649 | 2746 | 3.6E-01 | 0.82 |
| TIMP-2 | 1772 | 1879 | 1304 | 1926 | 5543 | 1494 | 4.0E-01 | 0.65 |
| MIP-3 a | 2114 | 1264 | 1195 | 1260 | 1128 | 1043 | 4.0E-01 | 1.29 |
| IL-6 | 629 | 334 | 587 | 617 | 760 | 632 | 4.2E-01 | 0.75 |
| IL-8 | 55037 | 62123 | 63515 | 54760 | 47289 | 48050 | 4.2E-01 | 1.20 |
| Eotaxin-3 | 2417 | 1968 | 1999 | 2178 | 1463 | 1785 | 4.2E-01 | 1.19 |
| MIP-1b | 54923 | 62761 | 63886 | 55289 | 50610 | 52128 | 4.6E-01 | 1.15 |
| RANTES | 55141 | 62786 | 63867 | 55154 | 49985 | 51247 | 4.6E-01 | 1.16 |
| IGFBP-3 | 1569 | 1446 | 1369 | 1819 | 3073 | 1282 | 4.6E-01 | 0.76 |
| IGFBP-4 | 1674 | 1801 | 1693 | 1863 | 2847 | 1714 | 4.6E-01 | 0.83 |
| IP-10 | 5353 | 4885 | 5193 | 5462 | 6145 | 5559 | 4.6E-01 | 0.90 |
| NT-4 | 2572 | 1771 | 2039 | 2145 | 4901 | 2097 | 4.6E-01 | 0.75 |
| IL-1a | 1012 | 631 | 569 | 997 | 875 | 902 | 4.6E-01 | 0.77 |
| IL-13 | 54782 | 60856 | 62184 | 54743 | 49803 | 50965 | 4.6E-01 | 1.14 |
| SDF-1 | 4373 | 3710 | 4517 | 3676 | 3918 | 3844 | 4.6E-01 | 1.10 |
| IL-16 | 3306 | 3332 | 3383 | 3411 | 6704 | 3159 | 4.6E-01 | 0.80 |
| NT-3 | 3007 | 2068 | 2567 | 3319 | 2560 | 2737 | 4.6E-01 | 0.88 |
| TGF- b 2 | 2963 | 2211 | 2581 | 2999 | 7957 | 2062 | 4.6E-01 | 0.70 |
| IL-15 | 2106 | 1777 | 2193 | 1826 | 1793 | 1907 | 4.8E-01 | 1.10 |
| TNF-a | 2846 | 2889 | 2712 | 2816 | 3119 | 3227 | 6.0E-01 | 0.92 |
| Ck b 8-1 | 1813 | 1317 | 1767 | 2050 | 1855 | 1645 | 6.0E-01 | 0.88 |
| FGF-9 | 2909 | 2513 | 2572 | 2884 | 3126 | 2890 | 6.0E-01 | 0.90 |
| TGF- b 3 | 2959 | 2485 | 2411 | 3316 | 2737 | 2839 | 6.0E-01 | 0.88 |
| IL-7 | 93 | 0 | 0 | 0 | 78 | 97 | 6.2E-01 | 0.23 |
| BDNF | 4016 | 3671 | 4210 | 4538 | 4245 | 4307 | 6.2E-01 | 0.91 |
| Osteopontin | 2691 | 2176 | 2084 | 2588 | 2610 | 2636 | 6.4E-01 | 0.88 |
| IL-4 | 682 | 707 | 720 | 752 | 740 | 837 | 6.6E-01 | 0.91 |
| PDGF-BB | 1390 | 1232 | 1209 | 1577 | 1262 | 1427 | 6.6E-01 | 0.90 |
| FGF-7 | 1506 | 1197 | 1674 | 1676 | 1976 | 1291 | 6.6E-01 | 0.89 |
| IL-1 b | 2965 | 2747 | 2757 | 2473 | 2722 | 2823 | 7.0E-01 | 1.06 |
| GDNF | 2741 | 2077 | 2336 | 2532 | 2897 | 2286 | 7.1E-01 | 0.93 |
| IGF-I | 1613 | 1910 | 1499 | 1692 | 1535 | 1422 | 7.2E-01 | 1.08 |
| FGF-6 | 2131 | 1908 | 2244 | 2421 | 2381 | 1914 | 7.2E-01 | 0.94 |
| Flt-3 Ligand | 3966 | 3988 | 4464 | 3809 | 4254 | 3631 | 7.2E-01 | 1.06 |
| IGFBP-1 | 3234 | 2445 | 2097 | 2783 | 2059 | 2323 | 7.2E-01 | 1.08 |
| TIMP-1 | 14980 | 12996 | 12630 | 14820 | 13094 | 15819 | 7.2E-01 | 0.93 |
| IL-3 | 2848 | 2781 | 2848 | 3240 | 3025 | 2697 | 7.7E-01 | 0.95 |
| IL-12 p70 | 2493 | 2250 | 2504 | 2375 | 2138 | 2415 | 7.7E-01 | 1.05 |
| TGF-b1 | 1058 | 902 | 941 | 1134 | 950 | 945 | 7.7E-01 | 0.96 |
| FGF-4 | 2799 | 2631 | 2682 | 2880 | 3490 | 2405 | 7.7E-01 | 0.93 |
| IGFBP-2 | 4474 | 3970 | 3892 | 4452 | 4261 | 4056 | 7.7E-01 | 0.96 |
| LIGHT | 2969 | 2747 | 2839 | 4346 | 2789 | 2397 | 7.7E-01 | 0.93 |
| Osteoprotegerin | 2118 | 1976 | 1953 | 2231 | 2068 | 2068 | 7.9E-01 | 0.95 |
| Eotaxin-2 | 2549 | 3089 | 2515 | 2368 | 3057 | 2430 | 7.9E-01 | 1.04 |
| PIGF | 1696 | 1543 | 1608 | 1434 | 1796 | 1423 | 8.3E-01 | 1.05 |
| BLC | 1767 | 1950 | 1973 | 2093 | 2552 | 1482 | 8.4E-01 | 0.95 |
| IL-10 | 3639 | 3474 | 1942 | 3353 | 3491 | 3691 | 8.6E-01 | 0.83 |
| VEGF | 2264 | 1843 | 2200 | 1985 | 2152 | 2005 | 9.4E-01 | 1.02 |
| SCF | 2192 | 1780 | 2153 | 1881 | 2101 | 2044 | 9.5E-01 | 1.01 |
| GCP-2 | 2517 | 2065 | 2108 | 2169 | 2277 | 2085 | 9.5E-01 | 1.02 |
| ENA-78 | 2447 | 2294 | 2503 | 2451 | 2244 | 2600 | 9.7E-01 | 0.99 |
| GRO-a | 1766 | 2129 | 1911 | 1678 | 1911 | 2295 | 9.7E-01 | 0.99 |
| MIP-1d | 615 | 498 | 611 | 518 | 590 | 646 | 9.7E-01 | 0.98 |
| Fractalkine | 2836 | 2274 | 2434 | 2498 | 2733 | 2348 | 9.7E-01 | 0.99 |
| Eotaxin | 2858 | 3307 | 2629 | 4259 | 2345 | 2535 | 9.8E-01 | 0.99 |
| MIF | 7059 | 6338 | 6400 | 6897 | 9278 | 4391 | 9.8E-01 | 1.01 |
